# The Neurorobotics Platform Robot Designer: Modeling Morphologies for Embodied Learning Experiments

**DOI:** 10.3389/fnbot.2022.856727

**Published:** 2022-04-25

**Authors:** Benedikt Feldotto, Fabrice O. Morin, Alois Knoll

**Affiliations:** Robotics, Artificial Intelligence and Real-Time Systems, Faculty of Informatics, Technical University of Munich, Munich, Germany

**Keywords:** simulation, neurorobotics, design, biomimetic robots, muscles, biomechanics, embodied AI

## Abstract

The more we investigate the principles of motion learning in biological systems, the more we reveal the central role that body morphology plays in motion execution. Not only does anatomy define the kinematics and therefore the complexity of possible movements, but it now becomes clear that part of the computation required for motion control is offloaded to body dynamics (a phenomenon referred to as “Morphological Computation.”) Consequentially, a proper design of body morphology is essential to carry out meaningful simulations on motor control of robotic and musculoskeletal systems. The design should not be fixed for simulation experiments beforehand, but is a central research aspect in every motion learning experiment that requires continuous adaptation during the experimental phase. We herein introduce a plugin for the 3D modeling suite Blender that enables researchers to design morphologies for simulation experiments in, particularly but not restricted to, the Neurorobotics Platform. We include design capabilities for both musculoskeletal bodies, as well as robotic systems in the Robot Designer. Thereby, we hope to not only foster understanding of biological motions and enabling better robot designs, but enabling true Neurorobotic experiments that may consist of biomimetic models such as tendon-driven robot as a mix of both or a transition between both biology and technology. This plugin helps researchers design and parameterize models with a Graphical User Interface and thus simplifies and speeds up the overall design process.

## 1. Introduction

The term Morphological Computation (Müller and Hoffmann, 2017) describes the principle of computations required for motion execution that are not implemented in a dedicated (electronic) controller but executed by the kinematics and morphology of the body itself. A well studied example for morphology facilitating control can be found in the passive dynamic walker (McGeer, 1990; Müller and Hoffmann, 2017), that locomotes smoothly down a slope without any computational unit and no energy input except gravitation. This principle posits that body morphology not only contributes to the execution of motions, but also that body design is a crucial determinant of behavioral capabilities of a given agent. Furthermore, several studies suggest that the morphological design may even have a decisive effect on cognition and hereby may influence thinking and problem solving (Pfeifer, 2000; Pfeifer and Bongard, 2006). At the very least, kinematics, dynamics, and geometry / anatomy enable or constrain actions that an agent is able to execute, as they mediate any intelligent interaction of an agent with its surrounding world. In a robotic view, the kinematic structure defines the workspace, actuators and sensors enable interaction with the environment in terms of action and perception.

The aforementioned constraints, computations, and influence of the morphology that impact the overall agent capabilities emphasize the importance of proper and thoughtful body design for (energy-) efficient and functionally capable agents. Therefore, we herein introduce the Robot Designer, a plugin for the 3D modeling suite Blender (Community, 2018) to facilitate the design of musculoskeletal, as well as robotic body models for simulation-based experiments. We propose a graphical user interface (GUI) with a range of tools for kinematics, dynamics, geometries, sensors, and muscles to promote easy and fast design and parameterization of agent bodies. Additionally, environmental setups can be generated. Models can be exported/imported in community standard formats such as SDFormat and .osim and are directly compatible with the Neurorobotics Platform (NRP) as a framework for embodied motion learning experiments. With the Robot Designer we introduce the first morphology design tool that integrates capabilities for both biological and robotic morphologies.

The Robot Designer is an ongoing Open Source project published under GPLv2 license in the BlenderRobotDesigner repository ^1^ of the Neurorobotics group in the Human Brain Project. Research in Artificial Intelligence and neural learning in the brain can largely benefit from this plugin as it helps building virtual environments for training data collection in simulation as well as opening the possibility to connect *in silico* brain models to musculoskeletal bodies, that cannot be built in the physical world, respectively.

In this article, we will introduce the State of the Art in robot design tools, and the Neurorobotics Platform as the central simulation as a prominent example on Neurorobotics simulation suite. We will then introduce the architecture and workflow building robot models with the Robot Designer before we go in depth into the various design steps and capabilities. Finally, we will present several examples of models created with the help of our plugin including robotic, musculoskeletal and biomimetic systems.

## 2. State of the Art

Simulation of the physical world has become ever more sophisticated in the past years in terms of computation of complex kinematic chains, computation performance for large 3D environments and photorealistic rendering. Improvements in simulation software have been matched by a strong increase in computational power available locally or on demand in highly parallel cloud computing instances. Robotic Machine Learning applications requiring large amounts of training data have grown rapidly and therefore robot simulation has attracted great interest while simultaneously stimulating the expression of many requirements.

Several simulation suites exist that are build for dedicated purposes; a comprehensive review of physics simulators can be found in Collins et al. (2021). The design and parameterization of virtual agents and environments, however, remains a tedious and time-consuming process. This is one reason why models and environments are usually provided within benchmark experiments and often are not modified by the actual users after release. Popular examples can be found in the field of Artificial Intelligence with the OpenAI gym environments (Brockman et al., 2016) and the NIPS learning to run challenge (Brockman et al., 2016).

Working with predefined models and environments eliminates the ability for users to exploit the full potential of intelligent body design that itself supports fast and efficient learning. In order to offer guided model design and easier parameterization while at the same time enabling a co-design process of morphology model along with the cognitive module, user-friendly design tools are necessary.

In this chapter, we introduce existing tools and conventions for simulation model design that we utilize for the Robot Designer or are related to our work. We also present the Neurorobotics Platform as an embodied learning suite wherein our exported models can be used.

### 2.1. Related Tools

The most basic and yet popular approach for model parameterization still is editing respective values in the model XML description. In order to generate a new model from scratch, one usually uses mesh files (commonly .dae or .stl file format) that originate from CAD software (e.g., CATIA, Autodesk, SolidWorks), or mesh generation with 3D modeling tools such as Blender and Maya, or other editing tools that specialize on specific model types (e.g., MakeHuman for human avatars). For some CAD design tools the community has developed plugins that support SDF or URDF (can be converted to SDF using Gazebo) export functionalities [e.g., SDFusion for AutoDesk Fusion 360 (Roboy development team, 2021), FreeCAD RobotCreator Workbench (Fosselius, 2017) and sw_urdf_exporter for SolidWorks (StephenBrawner, 2021)]. Most of them are inofficial though, and parameters that go beyond geometry and kinematics still need to be added manually later on. Many simulators include GUI elements to inspect or edit model parameters (e.g., Gazebo, Opensim). Gazebo also includes a dedicated model editor to build up models using existing mesh files or adapting existing models. Usually, a mix of manual XML editing, use of specialized tools for mesh editing, and use of available simulator user interfaces is utilized, and users build up their own custom pipelines with a mix of preferred tools.

The tool most similar to our plugin is the Phobos project (von Szadkowski and Reichel, 2020), that combines robot model parameterization, as well as sophisticated mesh editing in a single framework and is also implemented as a Blender plugin. The graphical user interface is optimized for robotic design, users can adapt all relevant parameters *via* GUI elements, and models can be exported/imported in URDF, SDF, and SMURF format. Phobos implements similar functionalities as the Robot Designer, the main differences being that we split the GUI in multiple tabs that represent and hereby structure the design process, as well as our focus on Neurorobotic models with support for musculoskeletal simulations.

The first iterations of the NRP RobotDesigner were initially developed under the name OpenGRASP RobotEditor (Leon et al., 2010) from 2008-2012 at the Humanoids and Intelligence Systems Lab (HIS) at the Institute for Anthropomatics and Robotics (IAR) of the Karlsruhe Institute of Technology in the context of the European GRASP project (Leon et al., 2010) as part of the OpenGRASP software.

The RobotEditor software focused on robotic design capabilities, as the support for valid models in Collada v1.5 data 3D asset exchange format had large support from the industry. It was later rewritten to comply with the programming interface of newer Blender versions (>2.69); support for sensors matching motion capture data for the Simox robot simulator (Vahrenkamp et al., 2012) was also added. The Robot Designer was initially forked from the RobotEditor project. Many features were added or optimized; in particular its main focus was shifted from pure robotic to Neurorobotic model design and direct compliance with the NRP.

### 2.2. Blender

**Figure 1:**
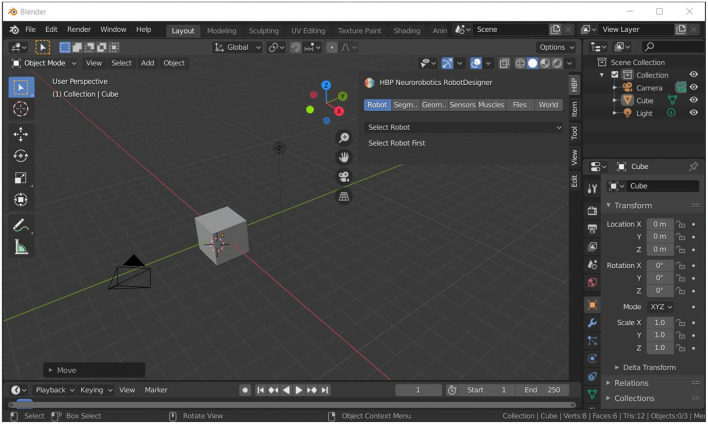
Graphical User Interface (GUI) of the 3D modeling suite Blender. The Robot Designer is implemented as a plugin to extend the design tools for robot and musculoskeletal modeling, specifically it can be expanded from the menu as shown in the top-right area of the figure.

Blender (Community, 2018) is an open source and feature rich suite for 3D modeling licensed under GPL. It includes tools for modeling, animation, rendering, compositing and motion tracking, video editing and 2D animation. Additionally, models can be rigged and it integrates a physics engine for simulation. A major strength of Blender is its open Python programming interface with integrated terminal and scripting that allows users to automate model design and easily write custom scripts and plugins. Blender provides all necessary tools for 3D mesh design suitable for Neurorobotic models. The Robot Designer extends the build in datatypes for robot and musculoskeletal modeling and its import and export. The plugin is integrated into Blenders plugin framework and is regularly updated to newer blender software versions, currently Blender version 2.82. Figure 1 shows the Graphical User Interface of Blender 2.82, on the right you can see the expanded Robot Designer Plugin that is subdivided into multiple tab sessions.

### 2.3. SDFormat

The Simulation Description Format (SDF) (Open Source Robotics Foundation, 2020) is a standardized XML description for models and environments in 3D simulations. It is specifically designed for robotic simulations that include environments, objects, and robot actors. The description format specifies kinematics, dynamics, and geometries of models that can be extended with sensors, actuators, and animations. General properties of the physics engine, environmental conditions, such as gravity and lighting and many others parameters can be specified; one or multiple passive objects and active robotic agents can be integrated. Mesh configurations can be linked as Collada (.dae) or STL files. SDFormat is particularly used for, but not limited to, the robotic simulator Gazebo (Koenig and Howard, 2004) that is widely used in the robotic community.

### 2.4. The Neurorobotics Platform

The Neurorobotics Platform (Knoll et al., 2016; Falotico et al., 2017; Albanese et al., 2020) is a simulation framework for embodied neural simulations that is developed in the framework of the Human Brain Project. At its core, spiking neural networks can be interconnected *via* so-called Transfer Functions with robotic or musculoskeletal simulation models interacting in virtual environments. With this approach, learning in neural networks can be studied in the context of a closed Perception-Cognition-Action loop (Vernon et al., 2015). The open source platform itself is built upon existing community tools, such as NEST (Gewaltig and Diesmann, 2007) for spiking neural networks, Gazebo (Koenig and Howard, 2004) and OpenSim (Delp et al., 2007) for body physics simulation, and ROS (Stanford Artificial Intelligence Laboratory et al., 2021) as communication interface. The platform can be installed locally but is also offered as a service on supercomputing cluster resources *via* browser login. Overall the platform aims to support understanding of embodiment for neural learning. Biologically plausible or artificial neural networks can be easily interfaced with musculoskeletal systems and robotic models, thereby supporting the study of transfer learning from biological to artificial agents and vice versa. The platform comes with a set of tools to implement, run and analyze embodied learning experiments: Robot and Environment Designer, browser-based Graphical User Interface for interactive experiment execution, plotting and spike train widgets for analysis, as well as a Virtual Coach for scripted (batch) experiment execution.

The Robot Designer has been introduced as a tool alongside this suite of tools within the NRP in (Falotico et al., 2017) in order to design and optimize simulation models. Since its introduction many improvements and adaptations have been made, in particular the support for biomimetic and musculoskeltal models.

In this article, we describe the Robot Designer in detail and introduce the evolved architecture, workflow and tools. Our plugin has been tested with NRP version 3.1 and will be continuously adapted for compatibility with future versions.

## 3. Plugin Architecture

**Figure 2:**
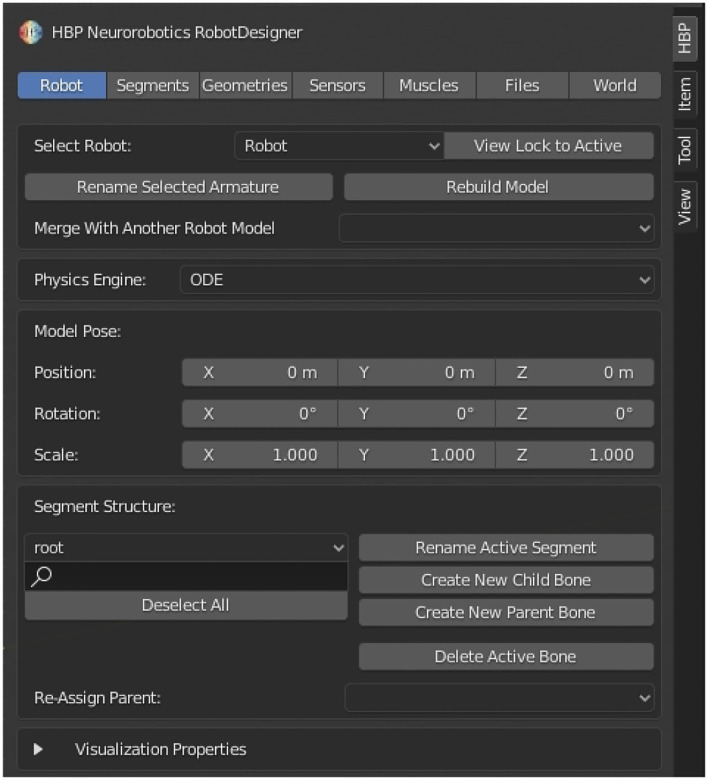
Robot Designer Graphical User Interface: The user interface is subdivided into separate sections, which guide the user through the dedicated steps in the design process and can be selected *via* tabs on the top line. Sections include: Robot, Segments, Geometries, Sensors, Muscles, Files, World.

The Robot Designer is implemented as a plugin in the 3D modeling suite Blender. Thereby, the capabilities of Blender can be utilized in order to design, e.g., geometries and add textures. At the time of writing of the present report, the supported version is Blender 2.8; continuous improvements will adapt the plugin for future releases. The installation instructions and a respective installation script for the Robot Designer in Blender can be found in the Readme description of the referenced GitHub repository. Descriptions in this article refer to the Robot Designer plugin version 3.2, additional screenshots from Robot Designer 3.1 (Blender 2.7) demonstrate the consistent workflow we establish with ongoing plugin development. With the Robot Designer models can be extended with robot and musculoskeletal specific characteristics. Properties include defining kinematics, dynamics and geometries, adding sensors and muscles and importing/exporting models in simulator-specific formats. With this integrative setup, users have the ability to build models from scratch by designing meshes and then building up the kinematic structure within a single framework. Figure 2 shows the graphical user interface of the Robot Designer Plugin. The user is guided through the design process by section tabs on the top from left to right. The Robot Designer is built upon Blender built-in functionalities and parameters where possible. In particular, it uses the built in rigging functionalities and extends and adds parameters as well as tools where necessary.

The plugin itself is structured into the following sub-folders, as it has been introduced in the RobotEditor project:

- core.
- export.
- interface.
- operators.
- properties.
- resources.

The listed components contain: Implementations for plugin registration (“core,”) import and export functionalities (“export,”) the graphical user interface (“interface,”) operator functions that implement the various design tools of the Robot Designer (“operators,”) global and specific parameters (“properties,”) as well as additional resources (such as a created logfile) (“resources.”) Generally, the plugin is separated in frontend (interface) and backend (operators) functionalities. Inside the “properties” folder, global properties, as well as separate files holding properties specific to objects and segments exist. Properties are registered to the scene (*global) or to dedicated Blender objects, respectively. Hereby, existing Blender objects are extended in our plugin with parameters specific to robot or musculoskeletal modeling.

The Robot Designer “core” implements a general plugin manager that is responsible for registration of the plugin in Blender, as well as operators and properties to the Blender namespace. Additionally, it holds the general plugin configuration. The “core” is mostly original from the RobotEditor and implements base classes for the graphical user interface, in particular a collapsible box and info box template. Similarly, a base class for operators is implemented that adds pre- and postconditions implemented as decorator functions. Through this mechanism, proper function of operators is ensured, e.g. the given function is only executed if a specific object is selected that is to be modified e.g. an armature when editing the robot or a mesh editing a geometry. A property handler for single properties and property groups wraps factory functions, adding not only getter and setter functions but also search functionalities. Lastly, the core implements logging to a logfile, adding functions that retrieves and formats the callstack.

For the plugin we make use of as most Blender properties, types and data objects as possible. In many cases, Blender objects are utilized to maintain common 3D modification and visualization using the Blender tools. Re-using meshes and curves as Robot Designer objects we add a property to the object that contains a tag indicating our intended purpose and thus can easily search for and sort objects in the Robot Designer.

## 4. Model Design Workflow

**Figure 3:**
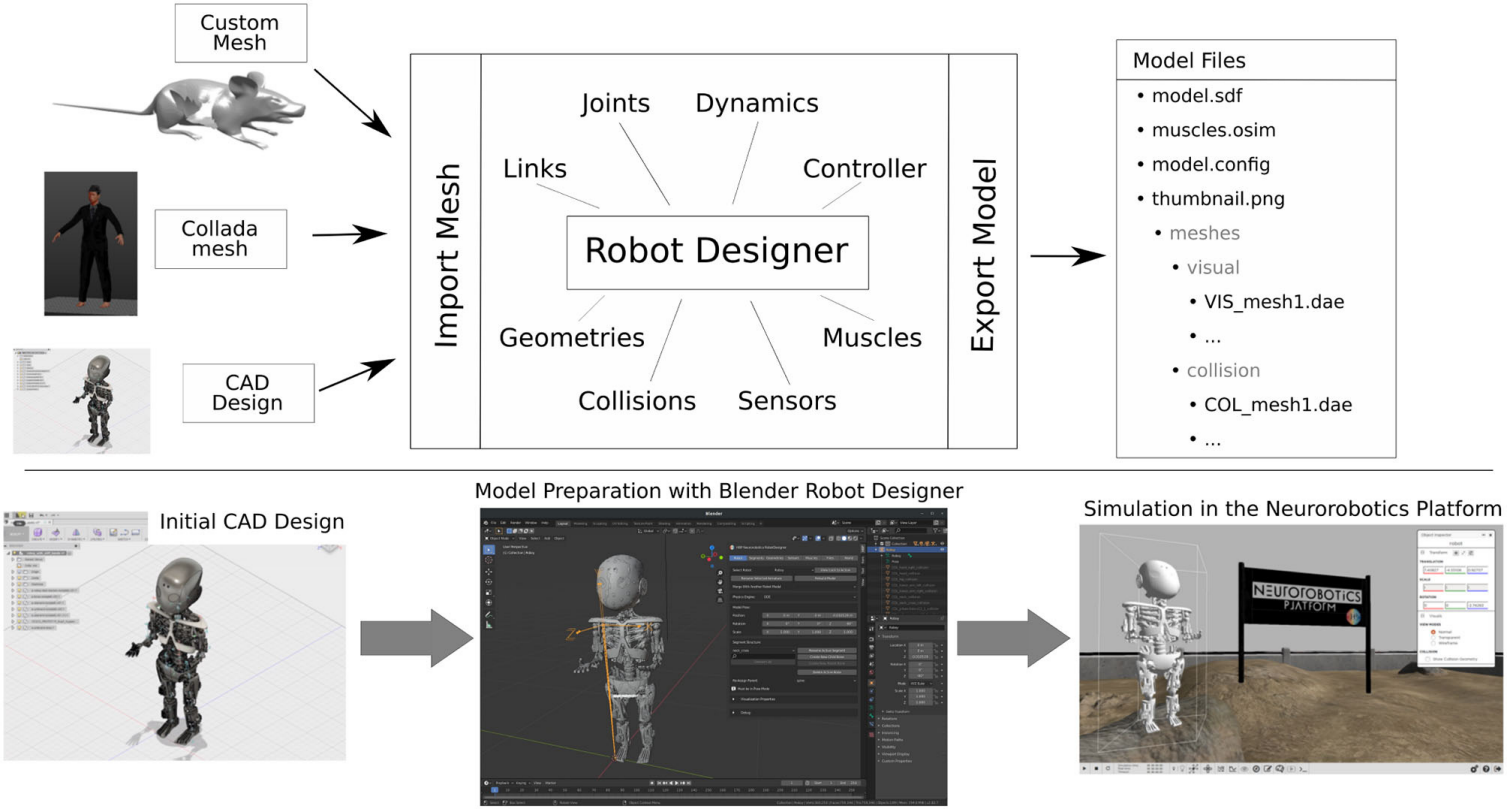
Robot Designer Workflow: The user can create a new model either by designing custom meshes from scratch or by importing an existing geometry, e.g., from CAD software. With the Robot Designer, all specifications relevant for robots and musculoskeletal models can be added and modified, ranging from kinematics, dynamics and geometries to sensors and muscles. Finally, the model description files are exported. The bottom part visualizes an exemplary workflow from CAD design *via* model preparation with the Robot Designer in Blender and finally its simulation in the Neurorobotics Platform.

With the Robot Designer, existing robot models can either be imported and adapted, or new models fully created from scratch. The general workflow is depicted in Figure 3 and is implemented in various tabs of the graphical user interface: users are guided through the design process and can walk through it selecting the offered tabs from left to right. Models are created with geometry meshes that can be imported from e.g. CAD software or manually designed in Blender utilizing the numerous modeling tools available. By utilizing elements of the plugins Graphical User Interface, links, joints, dynamics, controllers (Section 5), geometries, collisions (Section 6), and sensors (Section 7) can all be defined. For musculoskeletal models, a dedicated muscle tab helps define and parameterize muscle objects (Section 8).

After adding model metadata in the “files” section of the GUI, a model folder can be exported of the model description (model.config), model definition according to SDFormat (model.sdf), muscle definition in OpenSim specification (muscles.osim), as well as the referenced collision and visual meshes. The robot model can be directly imported into the NRP and simulated; without muscles, the exported model can also be used in every simulator that can handle the SDF format.

**Figure 4:**
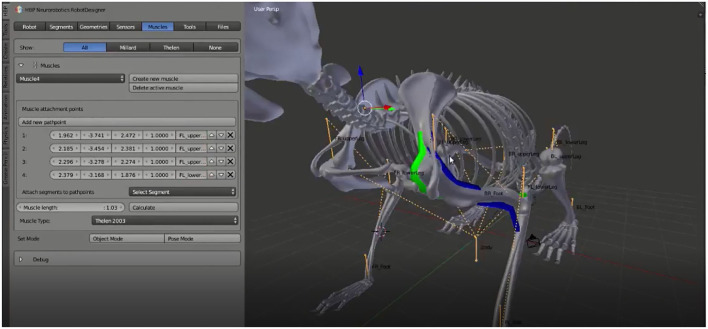
Musculoskeletal Model Design: Muscles are defined for a biological rodent model. Using the GUI and scene interaction, muscles can be specified with an arbitrary number of pathpoints by clicking the desired locations directly on the 3D model. All generated points are listed in the GUI and the exact location can be adjusted. Afterwards, muscle parameters such as muscle type, length and maximal force can be specified.

On the bottom part of Figure 4 a potential workflow is shown that goes from CAD design *via* Robot Designer parameterization to the final model simulated in the Neurorobotics Platfom. A video tutorial showcasing the design of a basic model as well as the graphical definition of muscles on a skeleton can be found in Feldotto (2017). Additionally, world files can be generated with environmental parameters for gravity and lighting, as well as one or multiple (robot) model instances (see Section 9).

## 5. Rigid Bodies

New model instances can be created, and existing instances selected, in the “Robot” tab. Here, the general 3D location, supported physics engine and the composition of links is defined. Afterwards, in the “Segments” section, kinematics, dynamics and controllers are specified as follows:

- **Kinematics**

Every model consists of one or multiple segments that are interconnected *via* fixed or moving joints. This kinematic chain can be configured either in Euler angles [*x, y, z*, α, β, γ] (Euler, 1968) or according to the Denavit-Hartenberg convention [θ, *d*, α, *a*] (Hartenberg and Denavit, 1964). Users can assign the desired joint type from a list of types such as fixed, prismatic or revolute, while static objects may be connected to the world with a fixed joint.

- **Dynamics**

Physics properties can be specified as mass, center of mass and inertia matrix individually for every segment. If the material making up the links can be taken to be homogeneous and its density can be provided, the center of mass and inertia can be calculated automatically based on the mesh geometry. Additional dynamic parameters include static and dynamic friction coefficients.

- **Controllers**

The Robot Designer supports PID controllers with proportional, integral and derivative parameters for every joint. These control parameters are exported as a general controller plugin shipped with the NRP in order to control every joint individually *via* ROS interface.

For every model selected, the kinematic chain is visualized as a linked graph in the 3D scene and inertia boxes can be visualized as translucent purple boxes, like in Gazebo. For the implementation of robotic links, we rely on the rigging functionalities of Blender: the kinematic chain is implemented as Blender armature (bpy.types.Armatures), that is extended with robot specific properties such as the Euler and Denavit-Hartenberg parameters. Inertia boxes are empty Blender objects for visualization and easy manipulation in the scene, again extended with the inertia matrix and weight properties.

## 6. Geometries

Next to the model kinematics, geometry plays an essential role, not only for visualization, but first and foremost in the computations of the interactions of a model with its surrounding environment. With the Robot Designer plugin all model geometries are implemented as Blender meshes that are tagged with an extended property in order to indicate their role for the given model. With the “Geometries” section tab the user can adjust the general properties for location, orientation, scaling and attach/detach geometries to the respective model links. For most physics engines, such as Gazebo, two separate geometries can be assigned per link. Collision meshes used for collision detection by the physics engine are typically a simplified version of the visual geometry; any increase in the complexity of these collision meshes entails an increase in computation time. The Robot Designers provides GUI buttons to enable easy addition of basic collision shapes (box, cylinder, sphere), as well as autogeneration of the convex hull of any visual geometry. Additionally, the vertex count per mesh can be reduced easily *via* a GUI button, for a single or all model meshes, in order to adjust the tradeoff of simplification and granularity for optimal physics engine performance and model behavior, respectively. In order to speed up the design process, many tools can be applied either to a single geometry or to all geometries of the selected model instance.

## 7. Sensors

Complementary to the control and actuation of body limbs, agents are endowed with perceptual capabilities through a variety of sensors. Here, the Gazebo and ROS communities offer a wide variety of sensor plugins that can be added to the model SDF description. Within the Robot Designer “Sensors” section, sensor instances can be added to the model graphically by clicking the desired 3D position in the scene. Afterwards the sensor is added to a specific model link, and sensor-specific parameters can be adjusted in the GUI. Camera sensors build up on Blender cameras, and are extended with specific properties that can be specified in the SDFormat. Since all sensor and controller plugins are specified as (ROS-) Gazebo plugins, new types and customized plugins can later be easily added in the introduced framework. Support for import and export of sensors and plugins will be implemented soon.

## 8. Muscles

The Neurorobotics Platform integrates the framework for muscle dynamics and pathpoint simulation from OpenSim into the “Simbody” physics engine in Gazebo in order to enable simulation of musculoskeletal systems and “classical” rigid-body robots within the same environment. Additionally, this integration allows the simulation of biomimetic robots that exhibit some degree of mechanical compliance, e.g., joints connecting rigid links but controlled with muscle-like actuators such as the Myorobotics framework (Marques et al., 2013). The Robot Designer provides graphical tools to easily define pathways and parameters for muscle or muscle-like actuators, thus enabling future research on biomechanics, soft robotics, etc.

### 8.1. Path and Attachment Points

An arbitrary number of muscles can be defined as a set of pathpoints (attachment points or *via*-points defining the muscle routing). Pathpoints are added by selecting a 3D position with the mouse cursor and approving with a GUI button. The exact location can afterwards be refined using the listed coordinates, and the connected link selected *via* drop-down menu. For this graphical manipulation, we implemented every muscle as Blender curve data object that is visualized in the 3D scene. Every muscle pathpoint is defined as a point on the underlying spline and can therefore be edited using the common 3D tools. As such, a muscle is a curve object, tagged with a custom property as a Robot Designer muscle and extended with specific properties such as muscle type and respective parameters to be modified in the GUI. An operator is implemented in order to calculate the length of the muscle automatically *via* a dedicated button.

Figure 4 shows muscles defined on a biological rodent skeleton, as well as the corresponding graphical user interface with generated muscle pathpoints and chosen parameters. Color coding indicates the various muscle types.

### 8.2. Wrapping Surfaces

OpenSim supports wrapping surfaces for muscles that do not follow a straight line but rather adapt and wrap to the skeletal surface. In the “muscles” section of the GUI, basic shapes (sphere, cylinder) can be added to the scene, attached to a segment and parameterized accordingly, whereas the process of mesh assignment to the body model can be done in a manner similar to basic collision geometries. Wrapping objects are Blender meshes tagged as wrapping object, they are color coded in the 3D view for visualization and graphical adaptation.

## 9. Worlds

In the study of embodied intelligence, the agent's body is important, but only inasmuch it enables interactions with the environment. In particular, by closing the action-cognition-perception loop, an agent can proactively change the environment with actions mediated by the body, thereby perceiving new sensory states the nature of which essentially depends on the environment. We, therefore, implemented a dedicated user interface section that enables the user to assemble environments by adding body models that can be either actuated robots or static objects such as a walls, shelves and books. Finally, the environment can be customized adding lights and adapting parameters such as gravity. This parameterization is particularly interesting for simulation to reality transfer of neural network applications, as randomization of such settings may cover a multitude of possible real world states. A Robot Designer world is implemented as a Blender empty object that sets the reference frame. It is extended with various parameters for the environment (e.g., gravity, wind), general physics engine parameters (e.g., max_step_size, real_time_factor, real_time_update_rate, max_contacts) and simulation engine (e.g., ODE, SimBody, OpenSim) specific parameters for contacts and friction. The world object holds a list of referenced models as custom properties.

## 10. Import and Export

Robot Designer models are particularly designed for, but not limited to, simulation in the Neurorobotics Platform; therefore, import/export functionalities are added accordingly. Models generated by the Robot Designer can be exported and imported in the SDFformat, linked geometries are exported as collada (.dae) mesh files. Technically, the exporter walks trough the armature from root to all branched links recursively and conglomerates the SDF specified parameters, both from the original Blender properties and from the custom Robot Designer properties that are assigned by the user. The SDFormat description does not itself contain an explicit graph description, but rather every link is defined with its parent and child. Therefore, importing a model from SDF first the link without parent or “world” as parent is found and then again recursively all child links are imported. As a result of this process, Blender objects are created in the 3D scene and the element specific properties are assigned to these objects. All geometries are exported in a dedicated mesh folder using the Blender Collada exporter and referenced in the SDF description. Additionally, where relevant, an OpenSim (.osim) file is generated holding the muscle description. For this purposes all muscle objects assigned to the given model are searched and exported as XML objects. Alongside every muscle, any referenced wrapping object is exported as well by adding its position, size and orientation parameters to the .osim description. Optionally, configurations for the ROS (tested with ROS Noetic) graphical user interface framework “RQt” (Thomas, Dirk and Scholz, Dorian and Blasdel, Aaron, 2016) can be exported. These include the configuration of plots for the rqt_multiplot package (Kaestner, Ralf, 2016), for sensory data (joint position, velocity, effort and/or muscle length, lengthening speed, force) as XML description file, and the YAML file description of GUI sliders provided by the rqt_ez_publihser package (Ogura, Takashi, 2016) in order to send commands to joint controllers and muscle actuators interactively. An additional model configuration file is generated for metadata such as author name and model description, and a thumbnail image showing the robot after applying Blender's rendering is exported alongside the model files.

The overall package of exported files describing rigid body or musculoskeletal models can be used directly in simulations running in the Neurorobotics Platform. Musculoskeletal models can also be simulated with the standalone Gazebo version shipped with the NRP that supports the OpenSim physics engine, models without muscles in standard Gazebo with different physics engines. Additionally, parts of the exported models might be used in other simulators as well. The exported SDFormat description of rigid body models can be simulated in any other framework supporting SDF, the .osim muscle description file can be reused in the OpenSim simulator. Model simulation has been tested with SDFormat 6.0 (SDF protocol 1.6) in Gazebo 9 and OpenSimDocument version 30000 supported by OpenSim 3.2, respectively.

## 11. Example Models

**Figure 5:**
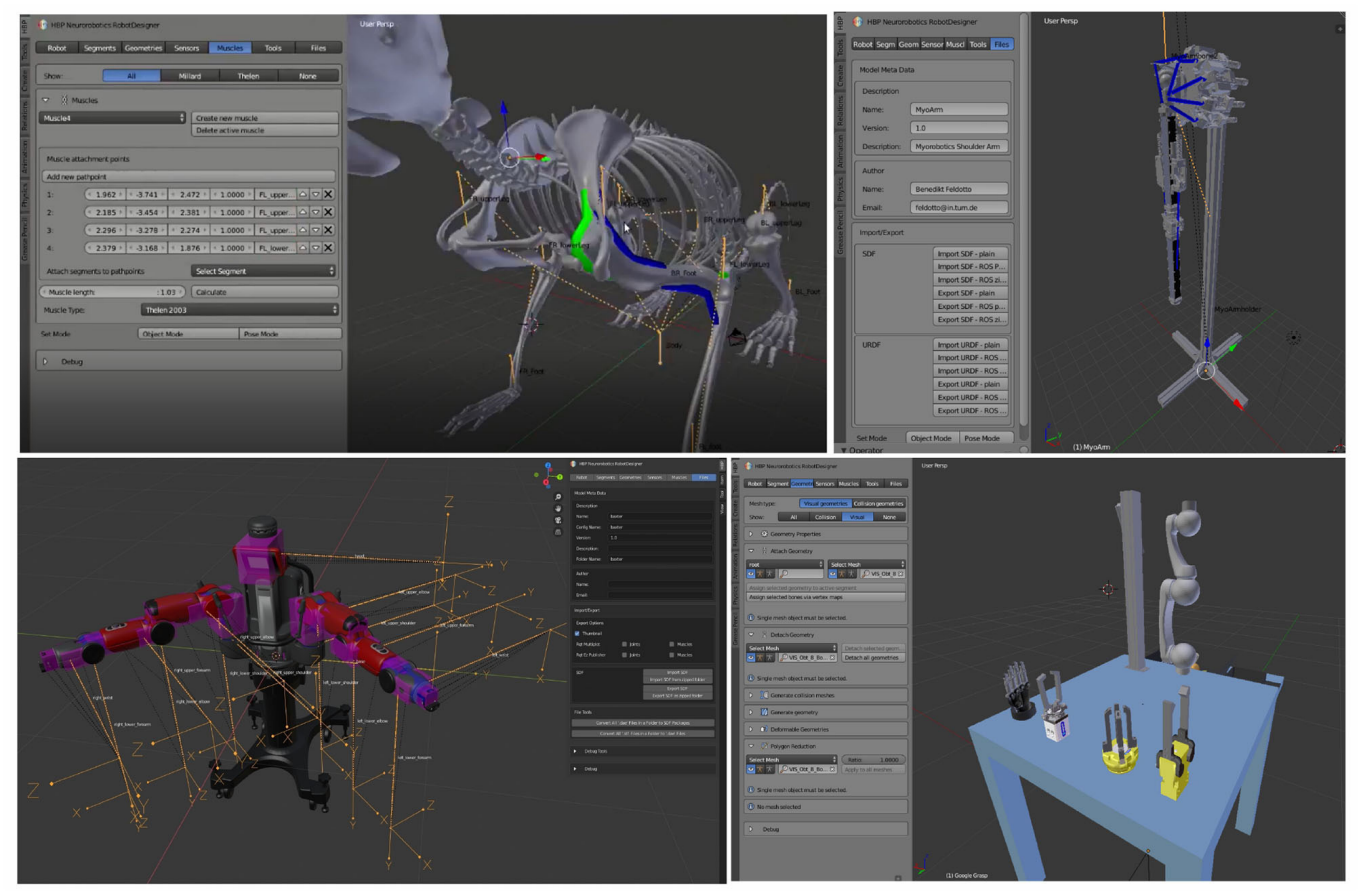
Example Robot Designer models: With the Robot Designer plugin, musculoskeletal models based on biological principles, or biomimetic robots exploiting biological characteristics, as well as classical robot models can be designed. From top left to bottom right: Musculoskeletal rodent model with skeleton and muscles, biomimetic robotic arm built from the modular Myorobotics toolkit with tendon actuators, humanoid robot Baxter, Schunk robotic arm with multiple grippers. In order to simplify the design process, visual cues are used and can be enabled optionally: orange lines indicate the kinematic tree, muscle colors indicate different muscle types, translucent purple boxes visualize the physics inertia. The user interface is kept consistent for Blender 2.7 and 2.8 (Baxter model).

The design capabilities provided by the Robot Designer plugin allow users to create a variety of simulation models. In particular, different model types can be created that range from classical robots, musculoskeletal models, and biomimetic robots as a mix of both. Figure 5 shows a selection of generated models that are generated or can be edited with our plugin: a musculoskeletal rodent model with several graphically defined muscles, a biomimetic robotic arm built with the Myorobotics toolkit and actuated with muscle-like tendon actuators, the humanoid robot “Baxter,” a Schunk robotic arm with different types of grippers.

**Figure 6:**
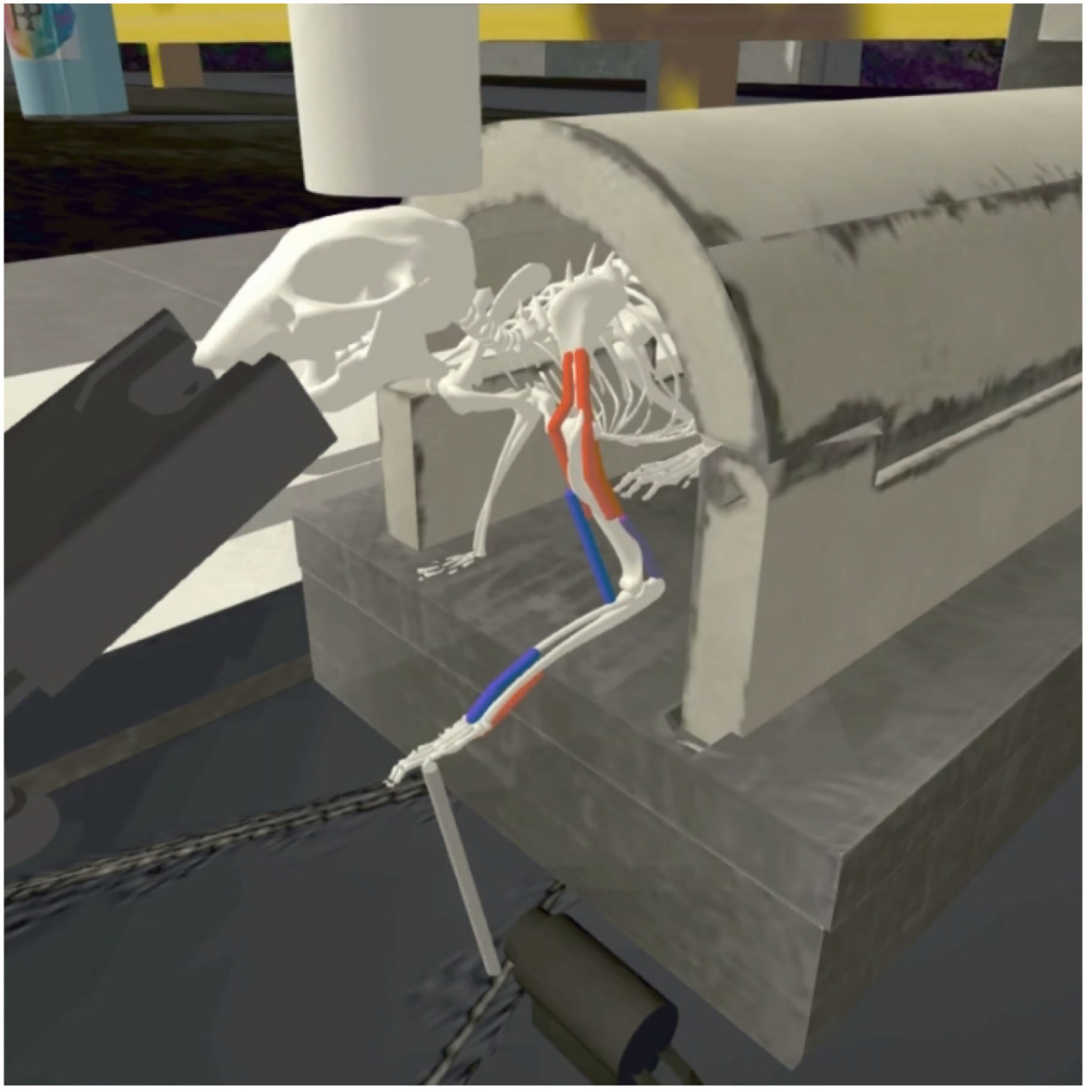
Model simulation in the Neurorobotics Platform: The musculoskeletal rodent model and experiment platform setup were modeled or adapted and aggregated with the Robot Designer. After exporting the model, it was simulated in the Neurorobotics Platform, and individual muscles could be controlled independently *via* ROS topics (active: red, inactive: blue).

Various additional color-coded 3D visualizations can be enabled throughout the design process. For example, colors indicate different muscle types on the rodent model, while the Baxter robot is visualized with its inertia boxes in translucent purple. The various design tools are aligned with simulation capabilities in the Neurorobotics Platform, and therefore form a strong asset for morphological design of agents for embodied simulation. Several models and environments available in the Neurorobotics Platform have been designed utilizing the Robot Designer plugin. As an example for the synergistic potential of both rigid body simulation fitted with muscles, in a recent paper (Mascaro et al., 2020) a rodent model has been set up using the Robot Designer for a stroke rehabilitation experiment. Figure 6 shows the rodent model adapted with additional muscles, a lickometer and an enhanced joystick controller for a 3D manipulation experiment, that were added using the Robot Designer. First, the kinematic structure and physical properties were defined, then muscle path and attachment points were designed and fine-tuned using the graphical user interface.The image shows the final simulation in the Neurorobotics Platform.

## 12. Conclusion

In this article, we have described the features of the Robot Designer plugin integrated into the 3D modeling suite Blender, which enables users to create and parameterize robot and musculoskeletal simulation models. In this process, users can exploit the capabilities for mesh editing of Blender, as well as robot- and muscle-specific features provided by our plugin in a single framework. We introduced tools for kinematic and dynamics definition, as well as mesh adaptation and optimization for both visual appearance and physics simulation. The plugin described herein provides a user-friendly and graphic centric approach for simulation model design. Its tool suite enables building better models, and also helps users to faster and more easily adapt model parameters for studying the impact of morphology on motion learning. The support of both robotic and musculoskeletal models in the Neurorobotics Platform and the Robot Designer enables users to investigate novel designs and control strategies for biomimetic robots that combine characteristics from both classical rigid robots and muscle-like actuators. The capabilities of the plugin for model export and import were specifically designed to align with the Neurorobotics Platform. However, because it relies on community-standard model description and muscle specifications, the Robot Designer may also be used outside of the ecosystem of the Neurorobotics Platform, with Gazebo or OpenSim standalone, for example. The Robot Designer code is open source (GNU GPL license) and the project will be continued considering user contributions and requests.

To the best of our knowledge, we here demonstrate the first modeling suite that integrates both robot and musculoskelal modeling in a single design framework. The support of muscle descriptions is a key feature that distinguishes our approach from existing tools such as the Phobos robotic design plugin. The graphical user interface aims to support a straightforward design process of simulation models, a process which still is often done by manually changing description files.

The Robot Designer can play an important role in current research fields of embodied neuroscience and biomimetic robots and will, therefore, be enhanced driven on future research requests. In future improvements, we will add support for sensor import/export and additional functionalities such as modularization with a library of morphology parts that can enhance but also hasten body design. Besides the improvement of the Robot Designer itself, further developments will also provide an enhanced interface to the Neurorobotics Platform which will allow scripted adaption of morphologies throughout experimental epochs, and/or experiments that generate optimized morphologies based on evolutionary development.

The Robot Designer can play an important role in current research fields of embodied neuroscience and biomimetic robots and will therefore be enhanced driven on future research requests. In future improvements, we will add support for sensor import/export and additional functionalities such as modularization with a library of morphology parts that can enhance but also hasten body design.

Besides the improvement of the Robot Designer itself, further developments will also provide an enhanced interface to the Neurorobotics Platform which will allow scripted adaption of morphologies throughout experimental epochs, and/or experiments that generate optimized morphologies based on evolutionary development.

## Data Availability Statement

Publicly available datasets were analyzed in this study. This data can be found at: GitHub, https://github.com/HBPNeurorobotics/BlenderRobotDesigner.

## Author Contributions

BF, FM, and AK conceptualized the Robot Designer extension for Neurorobotic modeling presented in this paper, building up on components of the predecessor RobotEditor, and wrote the paper. BF and FM orchestrated the implementation of the software project. BF is currently responsible for the Robot Designer code and user support, implemented the code for the new components, reviewed and integrated related code contributions, and build up most of the example models presented in this article. All authors contributed to the article and approved the submitted version.

## Funding

The predecessor RobotEditor was funded by the European Commission under the Information Society Technologies of the seventh Framework Programme (FP7). This project has received funding from the European Union's Horizon 2020 Framework Programme for Research and Innovation under the Specific Grant Agreements No. 720270 (Human Brain Project SGA1), No. 785907 (Human Brain Project SGA2), and 945539 (Human Brain Project SGA3).

## Conflict of Interest

The authors declare that the research was conducted in the absence of any commercial or financial relationships that could be construed as a potential conflict of interest.

## Publisher's Note

All claims expressed in this article are solely those of the authors and do not necessarily represent those of their affiliated organizations, or those of the publisher, the editors and the reviewers. Any product that may be evaluated in this article, or claim that may be made by its manufacturer, is not guaranteed or endorsed by the publisher.

## Footnotes

^1^ https://github.com/HBPNeurorobotics/BlenderRobotDesigner
